# Asthma diagnosis and treatment – 1022. The differences of clinical profiles by house dust mite sensitzation in patients with asthmatics in Soonchunhyang University Hospital cohort

**DOI:** 10.1186/1939-4551-6-S1-P21

**Published:** 2013-04-23

**Authors:** An-Soo Jang, Jong-Sook Park, Sung-Woo Park, June-Hyuk Lee, Dojin Kim, Choon-Sik Park

**Affiliations:** 1 Soonchunhyang University Bucheon Hospital, South Korea

## Background

The majority of patients with allergic disease are highly sensitized to house dust mites (HDM). There is few data to observe sensitization rate to HDM in asthmatics in Korea. The aim of this study was to observe the differences of clinical profiles by HDM sensitization in patients with asthmatics in Soonchunhyang University Hospital (SCH) cohort.

## Methods

We recruited 2316 asthmatic patients in SCH cohort. Lung function, BMI and sputum and blood eosinophils, and PC20, and clinical profiles were compared by HDM sensitization.

## Results

HDM sensitization rate was higher prevalence in male than in female. Compared with non-HDM sensitization asthmatics, HDM positive asthmatics had early onset of age (D. farina, 39.0 ± 0.50 vs. 48.5 ± 0.42, p=0.001; D. pteronyssinus, 39.4 ± 0.50 vs. 48.3 ± 0.43, p=0.001). HDM positive asthmatics had shorter duration of asthma symptom than that of HDM negative asthmatics. HDM negative asthmatics had lower FEV1/FVC % than those of HDM positive asthmatics. PC20 in HDM positive asthmatics had lower than those of HDM negative asthmatics (D. farina, 5.59 ± 0.25 mg/ml vs. 6.82 ± 0.28 mg/ml, p=0.001; D. pteronyssinus, 5.54 ± 0.24 mg/ml vs. 6.87 ± 0.28 mg/ml, p=0.001). Blood eosinophils number in D. pteronyssinus positive asthmatics had higher than that of D. pteronyssinus negative asthmatics (400.7 ± 12.5 vs. 373.5 ± 15.3, p<0.05). Total IgE in HDM positive asthmatics had higher than that of HDM negative asthmatics. There was no difference of BMI between two groups.

## Conclusions

Our data indicate that atopy asthmatics who sensitized to HDM had early onset of age, high total IgE and airway responsiveness, and eosinophilic inflammation.

